# Reconceptualizing school refusal behavior and related symptoms from an ecological perspective

**DOI:** 10.1002/ped4.70057

**Published:** 2026-04-06

**Authors:** Xiaoxuan Fan, Kui Wang, Tengteng Fan, Wanling Zhang, Aihua Wang, Chao Yan, Yanyu Wang, Ying Li

**Affiliations:** ^1^ Department of Psychosomatic Medicine, Beijing Children's Hospital Capital Medical University, National Center for Children's Health Beijing China; ^2^ State Key Laboratory of Cognitive Science and Mental Health, Institute of Psychology Chinese Academy of Sciences Beijing China; ^3^ Peking University Sixth Hospital Peking University Institute of Mental Health, NHC Key Laboratory of Mental Health (Peking University), National Clinical Research Center for Mental Disorders Beijing China; ^4^ School of Psychology and Cognitive Science East China Normal University Shanghai China; ^5^ School of Psychology Shandong Second Medical University Weifang Shandong China

School refusal behavior (SRB) has become a global and increasingly severe mental health and public health issue for children and adolescents. SRB is primarily an anxiety‐driven, non‐volitional pattern of avoiding or escaping school, distinct from willful truancy,^1^ with profound implications for their psychological and academic development. This phenomenon emerged before the pandemic and was exacerbated afterward. According to Australian data, the proportion of students achieving 90% attendance dropped from 77.8% to 59.8% between 2015 and 2024.^2^ Concurrently, a 2022 UK study reported that the persistent absence rate was 22.3%^3^; double the pre‐pandemic level. Globally, a growing number of students are struggling to adapt to returning to school, manifesting varying degrees of SRB. The United Nations Educational, Scientific, and Cultural Organization estimates that 10.9 million primary and secondary students worldwide are at risk of not returning to school following the pandemic.^4^ Addressing this issue necessitates establishing a multidimensional and multi‐tiered support system to better respond to the heterogeneity among children and adolescents with SRB.^5^

In China, according to the survey findings in 2016 by Chen et al., the incidence of SRB among primary and secondary school students in Guangzhou was approximately 22.5%.^6^ By 2024, a network analysis involving over 2000 students from high schools and vocational colleges in Guangzhou revealed that the incidence of SRB was 27.4%.^7^ These domestic findings suggest that, in the context of increasing social pressure, heavier academic burdens, and family dysfunction, a growing number of children and adolescents are facing the problem of SRB. Thus, SRB has become a global public health issue that transcends geographical and cultural boundaries, warranting urgent attention. However, SRB currently lacks a standalone diagnostic category in both the Diagnostic and Statistical Manual of Mental Disorders, Fifth Edition (DSM‐5) and the International Classification of Diseases, 11th Revision (ICD‐11) (with related symptoms classified under anxiety disorders). This diagnostic gap results in high heterogeneity in research and fragmented intervention approaches, due to the absence of an integrative conceptual framework.

## ETIOLOGY OF SRB: AN ECOLOGICAL PERSPECTIVE

Based on Bronfenbrenner's ecological systems theory, SRB is conceptualized as resulting from the accumulation of risk factors across interacting environmental systems, which leads to emotional, behavioral, and social dysfunction. Building on this framework and the key factors identified by Leduc et al.^8^ we conduct a contextualized analysis of the etiology of SRB.

Individuals with highly sensitive and perfectionistic traits are prone to excessive concern about interpersonal evaluation and low frustration tolerance. When encountering school‐related stressors (e.g., peer conflict and academic overload), these adolescents often resort to avoidance due to insufficient emotion regulation and adaptive coping strategies.^9^ SRB thus functions as a maladaptive short‐term mechanism for escaping stress and reducing anxiety. From a cognitive‐behavioral perspective, such behavior is closely linked to cognitive distortions in school‐related contexts, including catastrophic interpretations and negative self‐schemas, which directly provoke anxiety and depression.^10^ Refusal often serves to temporarily alleviate such emotional distress, making cognitive‐behavioral therapy a preferred intervention for SRB. Notably, these cognitive‐behavioral patterns do not form in isolation; erroneous beliefs in adolescents are correlated with family parenting styles. For instance, exposure to family violence increases the risk of cognitive distortions and involvement in school bullying.^11^

From a family systems perspective, SRB is linked to dysfunctional interaction patterns such as overprotection, high conflict, low support, and blurred boundaries, which correlate with adolescent distress.^9^ Parental emotional dysregulation—often tied to their own coping styles, academic values, and distress responses (e.g., permitting absence)—further destabilizes the family climate.^12^ This dysregulation impairs both individual role functioning and overall family stability, creating a self‐reinforcing cycle that undermines adaptation in other domains. Evidence consistently identifies dysfunctional family functioning as a key risk factor for SRB.^13^ Since microsystem elements interact continuously, cross‐system linkages (e.g., home‐school‐peer networks) are critical and must be integrated into individualized treatment plans.^1^

As the primary microsystem, the school system significantly influences SRB through social processes and academic structure.^8^ Socially, students often exhibit impairments in peer relationships, experience bullying or exclusion, lack a sense of belonging, and fear teacher interactions—challenges that hinder re‐entry and exacerbate distress. Academically, excessive workload and insufficient rest contribute to learning difficulties, cumulative pressure, and heightened fear of failure.^14^ During critical developmental stages, this chronic stress creates psychological conflict; many students reject not the content itself but the restrictive environment and monotonous format, revealing a mismatch between their needs and school reality.

Microsystems (family, school, and peers) are interdependent, forming the dynamically interconnected mesosystem. Understanding SRB thus requires tracing developmental trajectories, focusing on how interactions among these systems evolve and impact adaptive capacity, aligning with developmental psychopathology's emphasis on etiological factors within systemic interactions. Cross‐cultural research shows that integrated, multidisciplinary intervention models have emerged,^5^ affirming mesosystem theory's guiding value—it stresses that no single system (family, school, and healthcare) acts in isolation, highlighting instead the quality of inter‐system collaboration as the basis for building coordinated, multi‐level interventions. For example, Australia's “In2School” project integrates hospital, school, and family systems through a transition classroom where psychotherapists and teachers collaborate, employing strategies like cognitive behavioral therapy and graded re‐exposure to facilitate school return for anxious or depressed adolescents.^15^

Prevailing “meritocratic” societal pressures drive excessive familial expectations, internalizing as fear of failure and self‐worth instability, depleting psychological resources for school challenges, and forming a socio‐psychological basis for refusal. Concurrently, social media offers an immersive, instantly gratifying virtual space that substitutes for school‐based social and academic rewards.^16^ Major disruptions, such as a severe pandemic, have profoundly altered needs for safety and connection, disrupting school continuity and predictability. Prolonged isolation and reduced adaptability to collective settings further exacerbate avoidance, contributing to sustained rises in absenteeism.^3^

**FIGURE 1:**
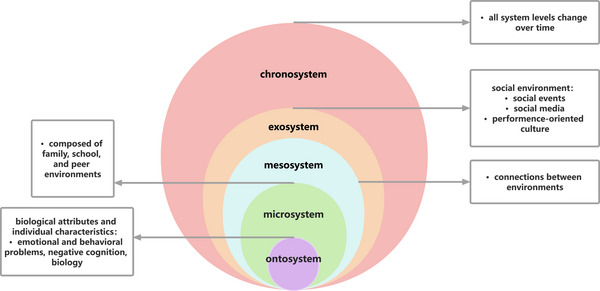
Etiological analysis of school refusal behavior within an ecological framework.

In summary, our etiological analysis of SRB from an ecological perspective (Figure 1) illustrates the contributory factors within this framework. SRB is the result of the interweaving, accumulation, and dynamic interaction of multiple risk factors across different levels, including the individual system, microsystems, the mesosystem, and the exosystem. Individual traits and cognitive biases constitute behavioral susceptibility, whereas dysfunction in microsystems such as family and school provides the proximal risk environment. Furthermore, the transfer of interaction patterns within the mesosystem and weak supportive linkages explain how problems are transmitted and amplified between systems, while broader external factors such as socio‐cultural pressures, the digital media environment, and major public events further shape the background context for the emergence of SRB. Ecological systems theory also emphasizes the role of the chronosystem, meaning that all system levels change over time, and an individual's adaptive pathway is likewise dynamic. Therefore, SRB is not an isolated phenomenon but a form of systemic maladjustment.

## DEFINITION AND CONNOTATION: PROPOSING THE CONCEPT OF “SCHOOL REFUSAL SYNDROME”

SRB warrants recognition as a distinct clinical entity. This is evidenced by its significant prevalence and functional impairment among adolescents,^2^ constituting a distinct public health concern; by its clinically complex presentation that often blends emotional, somatic, and behavioral issues, with a substantial proportion of cases not being fully accounted for by existing singular diagnoses like anxiety or depression^17^; by the notable gap in major diagnostic systems (e.g., DSM‐5 and ICD‐11), which fail to classify it as an independent condition, leaving many affected youths without accurate identification or targeted support^18^; by its fundamental nature as a transdiagnostic behavioral syndrome arising from the interaction of diverse psychopathological processes and environmental stressors^2^; and by the demonstrated practical need, as interventions solely targeting comorbid conditions are frequently insufficient, necessitating integrated strategies focused directly on school return and functional recovery.^19^ Therefore, it is essential to propose the conceptual framework of “school refusal syndrome” (SRS).​ This framework aims to fill the current diagnostic gap, providing a core construct that can encapsulate its complex manifestations, elucidate its transdiagnostic nature, and ultimately guide multi‐system clinical interventions.

The conceptualization of SRS as an operational diagnostic framework necessitates the integration of core clinical features, associated psychopathological indicators, and multidimensional assessment criteria. To meet the diagnosis of SRS, a child or adolescent must satisfy four mandatory core features: (A) Behavioral: A persistent, student‐motivated difficulty attending school—operationalized as an attendance rate below 80% over the past 2 weeks or below 50% over the past month—which is not primarily attributable to physical illness; (B) Emotional/cognitive: Significant school‐related distress manifested through intense anxiety, depressive symptoms, acute somatic complaints (typically peaking on school mornings), or a profound sense of meaninglessness regarding academic engagement; (C) Functional: Evidence of marked impairment in essential developmental domains, including academic progression, peer socialization, or family system stability; and (D) Temporal: A symptom duration of at least 2 weeks. Furthermore, the framework incorporates associated features such as problematic internet use, family conflict, or social withdrawal as key risk indicators. A comprehensive diagnosis requires a bio‐psycho‐social assessment to differentiate the syndrome's maintaining functions—such as escape from negative affect or pursuit of tangible out‐of‐school rewards—from comorbid conditions like clinical depression or anxiety disorders. Finally, the diagnosis necessitates the exclusion of pure truancy (characterized by parental unawareness and lack of emotional distress), primary psychotic disorders, and incapacitating physical diseases, thereby establishing SRS as a distinct, transdiagnostic behavioral construct.

The transition from SRB to SRS represents a vital conceptual evolution. Traditionally, early definitions of SRB were narrowly confined within the spectrum of anxiety disorders, often viewed merely as a symptomatic manifestation of separation anxiety or school phobia.^20^ In contrast, the SRS framework moves beyond this singular focus by reclassifying the phenomenon as a comprehensive clinical syndrome. Unlike the behavior‐centric SRB, SRS integrates multidimensional criteria—including emotional distress, cognitive dysfunction, and functional impairment—as core diagnostic components. By treating SRB as a transdiagnostic entity rather than a singular anxiety‐driven symptom, the SRS construct captures the complex interplay of diverse psychopathological and environmental factors, providing a more rigorous basis for targeted, multi‐systemic clinical intervention.

## CONCLUSION

This paper reconceptualizes SRB through ecological systems theory, proposing SRS as a psychopathology‐driven, persistent school attendance difficulty with significant functional impairments. SRS etiology stems from cumulative risks across nested systems: individual susceptibility (e.g., sensitivity and cognitive distortions); dysfunctional family/school microsystems; weak mesosystem linkages amplifying problems; and macrosystemic stressors (e.g., meritocratic pressure and digital immersion) that normalize avoidance. This necessitates a paradigm shift from individual‐focused to multisystem intervention. We propose an integrated intervention model coordinating four domains: family therapy to restore systemic functioning; school transformation to reduce threats and build competence; integrated pharmacotherapy for severe comorbidities; and community‐based practices to recalibrate digital‐skewed motivation. The goal is to break cross‐system avoidance cycles and rebuild adaptive pathways. By operationally defining SRS and outlining this ecological framework, we provide a coherent foundation for developing integrated, preventive support systems to address its multilayered roots.

## CONFLICT OF INTEREST

The authors declare no conflicts of interest.
